# A flexibly shaped space-time scan statistic for disease outbreak detection and monitoring

**DOI:** 10.1186/1476-072X-7-14

**Published:** 2008-04-11

**Authors:** Kunihiko Takahashi, Martin Kulldorff, Toshiro Tango, Katherine Yih

**Affiliations:** 1 Department of Technology Assessment and Biostatistics, National Institute of Public Health, Japan; 2 Department of Ambulatory Care and Prevention, Harvard Medical School and Harvard Pilgrim Health Care, Boston, USA

## Abstract

**Background:**

Early detection of disease outbreaks enables public health officials to implement disease control and prevention measures at the earliest possible time. A time periodic geographical disease surveillance system based on a cylindrical space-time scan statistic has been used extensively for disease surveillance along with the SaTScan software. In the purely spatial setting, many different methods have been proposed to detect spatial disease clusters. In particular, some spatial scan statistics are aimed at detecting irregularly shaped clusters which may not be detected by the circular spatial scan statistic.

**Results:**

Based on the *flexible purely spatial scan statistic*, we propose a flexibly shaped space-time scan statistic for early detection of disease outbreaks. The performance of the proposed space-time scan statistic is compared with that of the cylindrical scan statistic using benchmark data. In order to compare their performances, we have developed a space-time power distribution by extending the purely spatial bivariate power distribution. Daily syndromic surveillance data in Massachusetts, USA, are used to illustrate the proposed test statistic.

**Conclusion:**

The flexible space-time scan statistic is well suited for detecting and monitoring disease outbreaks in irregularly shaped areas.

## Background

The anthrax terrorist attacks in 2001, the severe acute respiratory syndrome (SARS) outbreak in 2002, and a concern about pandemic influenza have motivated many public health departments to develop early disease outbreak detection systems. Early detection of disease outbreaks enables public health officials to implement disease control and prevention measures at the earliest possible time. For an infectious disease, improvement in detection time by even one day might enable public health officials to control the disease before it becomes widespread. In many cities such as New York City [1], Washington, D.C. [2], Boston [3,4], Denver, and Minneapolis, real-time, geographic, early outbreak detection system have been implemented. For a well-defined geographical area, standard disease surveillance uses purely temporal methods that seek anomalies in time series data without using spatial information [5]. The increased need for geographical cluster detection has coincided with an increasing availability of spatial data [6]. Investigators ask whether the geographical cluster is unlikely to have arisen by chance given random variations from the background incidence, according for the multiple comparisons inherent in the many possible cluster locations and size evaluated. Scan statistics are tools to answer such questions [7,8]. Increasingly, there is interest in the prospective surveillance of new data as it becomes available in order to detect a localized disease outbreak as early as possible. Particularly in light of the perceived threat of bioterrorism and newly emerging infectious diseases, there has been a spate of recent interest in the development of geographic surveillance systems that can detect changes in spatial patterns of disease [9]. Recently, a time periodic geographical disease surveillance system based on a cylindrical space-time scan statistic was proposed by Kulldorff and colleagues [10,11].

Several different approaches to the statistical assessment of potential geographic clustering in either point-or area-based disease data have been developed [12,13]. Almost all of these purely spatial approaches are retrospective, in the sense that they describe statistical tests that are designed to be carried out once, on a set of data that has been collected from the recent past [9]. In particular, the circular spatial scan statistic [8] has been used extensively for the detections and evaluation of purely spatial disease clusters along with the SaTScan software [14]. For example, as part of their cancer surveillance initiative, the New York State Department of Health used the spatial scan statistic to look at the geographical variation of breast, lung, prostate, and colorectal cancer incidence in New York State, finding various statistically significant clusters but no local hotspots with greatly elevated risk [15]. However, as the statistic uses a circular scanning window with variable size to define the potential cluster area, it is difficult to correctly detect some non-circular clusters such as those along a river [16]. Recently, spatial scan statistics for irregular shaped clusters have been proposed, using the same likelihood ratio test formulation as before. The spatial scan statistics proposed by Duczmal and Assunção [17], Patil and Taillie [18], Tango and Takahashi [16], Assunção *et al*. [19] and Kulldorff *et al*. [20] are aimed at detecting irregularly shaped clusters which may not be detected by the circular spatial scan statistic. Due to the unlimited geometric freedom of cluster shapes, some of these statistics run the risk of detecting quite large and very peculiarly shaped clusters. The *flexible spatial scan statistic *[16], which has been used along with the FleXScan software [21], has a parameter *K *as the pre-set maximum length of neighbors to be scanned, to avoid detecting clusters with a very peculiar shape.

In this paper, we propose a flexibly shaped space-time scan statistic ("flexible space-time scan statistic" hereafter) for the early detection of disease outbreaks. It is based on the flexible purely spatial scan statistic [16] and the prospective space-time scan statistic [10]. The performance of our proposed space-time scan statistic is compared with that of the cylindrical scan statistic, using the benchmark data provided by Kulldorff *et al*. [22]. In order to evaluate its performance we propose a space-time power distribution by extending the purely spatial bivariate power distribution [16]. Daily syndromic surveillance data in Massachusetts, USA, are used to illustrate the proposed method with real data.

### The flexible space-time scan statistic

Consider the situation where an entire study area is divided into *m *regions (for example, counties, ZIP codes, enumeration districts, etcetera), and each region is periodically reporting the number of cases of a disease or syndrome under study. We assume that, under the null hypothesis of no clustering, the number of cases *N*_*id *_is a Poisson random variable with the observed value *n*_*id *_and the expected values *μ*_*id *_in each region *i*(*i *= 1,...,*m*) at time *d*, where *μ*_*id *_is proportional to its population size, or a covariate-adjusted population at risk. Since we are only interested in detecting clusters that are alive (active) at the current time *t*_*P*_, we only consider 'alive' clusters that are present in the following *T *time intervals:

[*t*_*P *_- *T *+ 1, *t*_*P*_], [*t*_*P *_- *T *+ 2, *t*_*P*_],..., [*t*_*P *_- 1,*t*_*P*_], [*t*_*P*_, *t*_*P*_]

where *T *is a pre-specified maximum temporal length of the cluster.

A time periodic geographical disease surveillance system based on a *cylindrical space-time scan statistic *has already been proposed by Kulldorff [10]. The cylindrical space-time scan statistic uses a cylindrical window in three dimensions where the base of the cylinder represents space and the height represents time. As with the purely spatial scan statistic, the cylindrical space-time scan statistic imposes a circular base *Z *on each centroid of regions for each of *T *time intervals. For each of centroids, the radius of the circle is varied from zero up to a pre-set maximum radius, for example, so that the window never includes more than 50% of the total population at risk [8]. In this paper, we use a pre-set maximum number of regions *K *to be included in the cluster as an upperbound of the radius. If the base contains the centroid of a region, then that whole region is included in the base. In total, a very large number of different but overlapping circular bases are created, each with a different set of neighboring regions and each being a possible candidate area containing a disease outbreak. Let *Z*_*ik*_, *k *= 1,...,*K*, denote the base composed by the region *i *and the (*k *- 1)-nearest neighbors to *i*. Then, all the cylindrical windows to be scanned by the cylindrical scan statistic are the cylinders with the base in the set

(1) $$Z_{1}=\{Z_{ik}|1\leq i\leq m,1\leq k\leq K\}$$

and the heights in the set

(2) $$Y=\{[t_{P}-t+1,t_{P}]|1\leq t\leq T\},$$

On the other hand, a *flexible space-time scan statistic *which we propose in this paper imposes a three dimensional prismatic window with an arbitrarily shaped base *Z*. For any given region *i*, we create the set of arbitrarily shaped bases consisting of *k *connected regions (1 ≤ *k *≤ *K*) including *i*. To avoid detecting a cluster of unlikely peculiar shape, the connected regions are restricted as the subset of the *K*-nearest neighbors to the region *i*, where *K *= 1 implies the region *i *itself. Let *Z*_*ik*(*j*)_, *j *= 1,...,*j*_*ik *_denote the *j*-th window which is a set of *k *regions connected starting from the region *i*, where *j*_*ik *_is the number of *j *satisfying *Z*_*ik*(*j*) _⊆ *Z*_*iK *_for *k *= 1,...,*K*. Then, all the windows to be scanned are the prisms whose base is included in the set

(3) $$Z_{2}=\{Z_{ik(j)}|1\leq i\leq m,1\leq k\leq K,1\leq j\leq j_{ik}\}$$

with height in the set Y. In other words, for any given region *i*, the cylindrical scan statistic consider *K *concentric circles for the base, whereas the flexible scan statistic consider *K *concentric circles plus all the sets of connected regions including the single region *i*, whose centroids are located within the *K*-th largest concentric circle.

Define *L*(*W*) as the likelihood under the alternative hypothesis that there is a cluster in the space-time window *W*(∈ W), where $W=Z_{1}\times Y$ (or $Z_{2}\times Y$) and *L*_0 _the likelihood under the null hypothesis. Then, conditioning on the observed total number of cases, *N*, the definition of the space-time scan statistic *S *is the maximum likelihood ratio over all possible windows *W*,

(4) $$S=\frac{\max_{W\in W}\{L(W)\}}{L_{0}}=\underset{W\in W}{\max}\left\{\frac{L(W)}{L_{0}}\right\}.$$

Let *n*_*W *_be the number of cases in window *W *. For the Poisson model, let *μ*_*W *_be the expected number in window *W *under the null hypothesis, so that *μ*_*G *_= *N *for *G*, the entire study space in three dimensions. It can then be shown that

(5) $$\frac{L(W)}{L_{0}}={\left(\frac{n_{W}}{\mu_{W}}\right)}^{n_{W}}{\left(\frac{N-n_{W}}{N-\mu_{W}}\right)}^{N-n_{W}}$$

if *n*_*W *_> *μ*_*W*_, and *L*(*W*)/*L*_0 _= 1 otherwise. The window for which the likelihood ratio is maximized identifies the most likely cluster (MLC) [8]. To find the distribution of the log likelihood ratio (LLR) under the null hypothesis, Monte Carlo hypothesis testing [23] is required. *p*-value of the test is based upon the null distribution of LLR with large number *B *of Monte Carlo replications of data sets generated under the null hypothesis, i.e.,

$$\hat{p}\frac{1+\sum_{v=1}^{B}I(LLR_{v}\geq LLR^{*})}{B+1}$$

where *LLR*_*v *_and LLR* is the value of the test statistic for the *v*-th Monte Carlo replicate and that for the observed data, respectively, and *I*(·) is the indicator function.

### Syndromic surveillance in Massachusetts

We applied the prospective flexible space-time scan statistic to daily syndromic surveillance data in eastern Massachusetts mimicking a real time surveillance system. The data came from an electronic medical record system used by Harvard Vanguard Medical Associates [3,24]. We used the rash and respiratory data during August 1–30, 2005. The data are geographically aggregated to ZIP codes. The number of ZIP codes used were different for each syndrome, for example cases of the rash were analyzed in 252 ZIP codes and respiratory in 385. Note that for the flexible space-time scan statistic, the ZIP code whose data does not exist, was treated like a ravine. For example, assume that ZIP codes *i*_1 _and *i*_2_, *i*_2 _and *i*_3 _are adjacent each other, respectively, but *i*_1 _and *i*_3 _are not adjacent. If the data of *i*_2 _does not exist under the situation, then it is assumed that *i*_1 _and *i*_3 _are not directly connected.

Based on the prior daily data for over a year in MA, the expected number of cases were calculated as the predicted means from a generalized linear mixed model (GLMM) as developed by Kleinman *et al*, adjusted for seasonal effect, day of week, etc, these are the same expectations used in the actual real time surveillance system [25]. We set *K *= 20 as the maximum length of the geographical window, and the maximum temporal length to be *T *= 7 days. The number of replications for the Monte Carlo procedure was set to *B *= 999. In disease outbreak detection, the recurrence interval (RI) is often used as an alternative to the *p*-value [14]. The measure reflects how often a cluster will be observed by chance, assuming that analyzes are repeated on a regular basis with a periodicity equal to the period of the study. For daily surveillance such as this analysis, the *p*-value of 0.001 corresponds to the RI of 1,000 days, i.e., 2.7 years, and an alpha level of 0.0027 corresponds to one expected false alarm every year.

**Figure 1:**
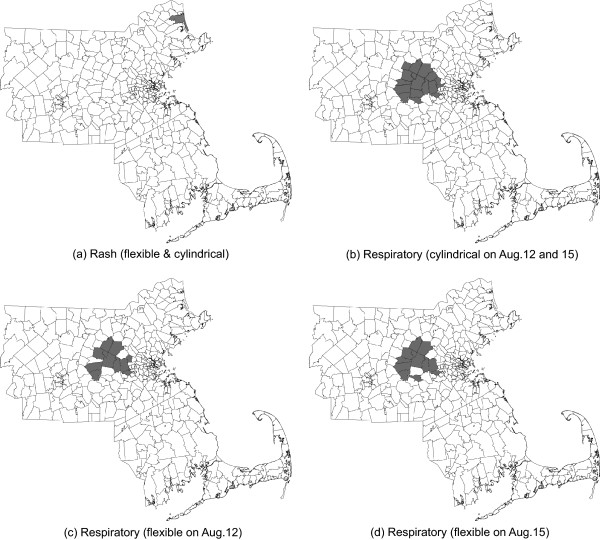
Detected outbreaks of Rash and Reepiratory in eastern Massachusetts during August 1–30, 2005, by the cylindrical scan statistic ((a) and (b)) and the flexible scan statistic ((a), (c) and (d)).

The results of analysis during August 1–30 by the flexible and the cylindrical space-time scan statistics are given in Tables 1, 2 and Figure 1. The tables show results for the days with *p *< 0.0054, which corresponds to the RI of at least 6 months. When looking at rash outbreaks (Table 1), both tests detected the same cluster with a single ZIP code 01951 on August 7, with the same temporal length (6 days) and the same RI (2.7 years). Note that the clusters detected by both tests from August 8 to 10 are not signals of an outbreak because the number of cases on August 8 must be 0, and on August 9 and 10, the number of cases of the cluster was decreasing. For respiratory syndrome (Table 2), each test detected a different cluster with the same RI of 2.7 years on August 12. The cluster detected by the flexible scan statistic contained 12 ZIP codes, while that from the cylindrical scan statistic contained 18 ZIP codes, with 11 ZIP codes detected in common. On August 13 and 14, the flexible scan statistic detected significant clusters with larger RIs, 333 days and 250 days respectively, while the cylindrical scan statistic detected clusters with short RIs, 91 days and 30 days respectively. The flexible scan statistic also detected a cluster on August 15 (RI = 1.4 years) with a temporal length of 6 days, while the cylindrical scan statistic detected a cluster with a temporal length of 5 days (RI = 200 days). For the 6 days from August 12 to 17 (results on August 16 and 17 are not shown in Table 2 because of shorter RIs), the cylindrical scan statistic kept detecting the same cluster, while the flexible scan statistic detected a similar but slightly different cluster each day. However, we should acknowledge the similar lack of evidence in Table 2 for a continued outbreak on August 13 to 14, because the number of additional cases on those days is very close to the expected number of additional cases. On the other hand, there is some evidence for an excess of cases on August 15 (23 additional cases), although the estimated relative risk is substantially reduced.

**Table 1:** Detected outbreaks of Rash based on daily syndromic surveillance data in eastern Massachusetts during August 1–30, 2005.

Day	zip codes	cluster period	cases	expected	llr	R.I.(*p*-value)
Rash:						
- flexible						
Aug.07	01951	Aug.02–07	7	0.0427	27.949	2.7 years(0.001)
Aug.08	01951	Aug.02–08	7	0.0545	26.259	2.7 years(0.001)
Aug.09	01951	Aug.03–09	6	0.0545	21.562	2.7 years(0.001)
Aug.10	01951	Aug.04–10	5	0.0545	17.315	2.7 years(0.001)
						
- cylindrical						
Aug.07	01951	Aug.02–07	7	0.0427	27.949	2.7 years(0.001)
Aug.08	01951	Aug.02–08	7	0.0545	26.259	2.7 years(0.001)
Aug.09	01951	Aug.03–09	6	0.0545	21.562	2.7 years(0.001)
Aug.10	01951	Aug.04–10	5	0.0545	17.315	2.7 years(0.001)

**Table 2:** Detected outbreaks of Respiratory based on daily syndromic surveillance data in eastern Massachusetts during August 1–30, 2005.

Day	zip codes	cluster period	cases	expected	llr	R.I.(*p*-value)
Respiratory:						
- flexible						
Aug.12	01720, 01742, 01752, 01754, 01772, 01775, 01776, 01778, 02451, 02462, 02481, 02493	Aug.11–12	42	12.452	17.635	2.7 years (0.001)
Aug.13	01720, 01742, 01749, 01752, 01754, 01772, 01775, 01776, 01778, 02451, 02462, 02481, 02493	Aug.11–13	46	14.950	16.634	333 days (0.003)
Aug.14	01720, 01742, 01749, 01752, 01754, 01772, 01775, 01776, 01778, 02451, 02462, 02481, 02493	Aug.11–14	49	16.957	15.927	250 days (0.004)
Aug.15	01702, 01720, 01742, 01749, 01752, 01754, 01772, 01775, 01776, 01778, 02481, 02493	Aug.10–15	72	29.975	16.726	1.4 years (0.002)
- cylindrical						
Aug.12	01701, 01702, 01718, 01719, 01720, 01742, 01749, 01752, 01754, 01772, 01773, 01775, 01776, 01778, 02451, 02453, 02481, 02493	Aug.11–12	51	20.036	12.688	2.7 years (0.001)
Aug.13	01701, 01702, 01718, 01719, 01720, 01742, 01749, 01752, 01754, 01772, 01773, 01775, 01776, 01778, 02451, 02453, 02481, 02493	Aug.11–13	55	23.768	10.945	91 days (0.011)
Aug.14	01701, 01702, 01718, 01719, 01720, 01742, 01749, 01752, 01754, 01772, 01773, 01775, 01776, 01778, 02451, 02453, 02481, 02493	Aug.11–14	59	26.959	10.221	30 days (0.033)
Aug.15	01701, 01702, 01718, 01719, 01720, 01742, 01749, 01752, 01754, 01772, 01773, 01775, 01776, 01778, 02451, 02453, 02481, 02493	Aug.11–15	82	40.981	11.662	200 days (0.005)

### Statistical power, sensitivity and positive predictive value

In this section, we compare the flexible and cylindrical space-time scan statistics, using benchmark data from 176 New York City ZIP codes ([14,22]). This benchmark data has been described in detail elsewhere [22], and here we only give a brief overview. Based on 2002 numbers, the total population is 8,003,510. The benchmark data sets contain a number of randomly located of cases of a hypothetical disease or syndrome, generated either under the null model with no outbreaks or under one of eight different alternative models with an outbreak in one of four different locations and with either a high or modest excess risk. For each of the null and alternative models, three different sets of data sets were generated, with 31, 32, and 33 days, respectively. For each of the null models, 9,999 random data sets were generated. For each of the alternative models, 1,000 random data sets were generated.

For each data set, the total number of randomly allocated cases was 100 times the number of days (i.e., 3,100 cases in the data sets containing 31 days). The number 100 was chosen to reflect the occurrence rate of certain syndromes common to the NYC emergency department(ED)-based syndromic surveillance system. Under the null model, each person living in NYC is equally likely to contract the disease, and the time of each case is assigned with equal probability to any given day. Thus, each case was randomly assigned to ZIP code *i *and day *d *with probability proportional to *μ*_*id *_= *pop*_*i*_, where *pop*_*i *_is the population of ZIP code *i*. For the alternative models, one or more ZIP codes were assigned an increased risk on Day 31 and, when applicable, on Days 32 and 33 as well. For these ZIP code and day combinations, *μ*_*id *_was multiplied by an assigned relative risk. For all other ZIP code and day combinations, *μ*_*id *_did not change. Each case was then randomly assigned with probability proportional to the new set of *μ*_*id *_to generate data under the alternative models.

**Figure 2:**
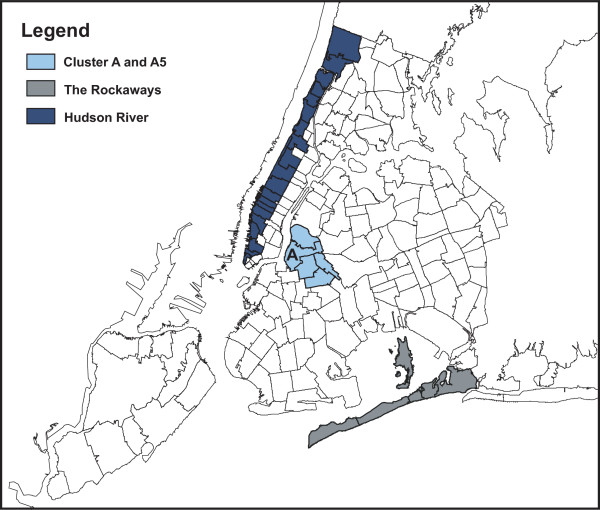
NYC 176 ZIP codes area and assumed clusters (i) Cluster A, (ii) Cluster A5, (iii) The Rockaways, and (iv) Hudson River.

Eight alternative models were evaluated, based on four different outbreak areas of length s* and total population pop* therein, with either high or medium relative risk (RR) [22] (Figure 2).

1. Cluster A: a single ZIP code area in Brooklyn (circular area)

s* = 1, pop* = 85, 089, RR: high = 9.91, medium = 5.66

2. Cluster A5: the same ZIP code with 4 neighboring ZIP codes (non-circular area)

s* = 5, pop* = 318, 754, RR: high = 4.47, medium = 3.06

3. The Rockaways, 5 ZIP codes area (non-circular area)

s* = 5, pop* = 106, 738, RR: high = 8.48, medium = 5.01

4. Hudson River: 20 ZIP codes areas along the shore of the Hudson River (non-circular area)

s* = 20, pop* = 827, 382, RR: high = 2.97, medium = 2.24

A maximum length of the geographic window *K *= 20 was used for the flexible scan statistic, while the cylindrical scan statistic used a maximum of either *K *= 20 or a 50 % of the population at risk. A period of *T *= 3 days was used as the maximum temporal length of the cluster. We did not use the options to include purely temporal clusters (see details in [14]).

### Standard statistical power

First of all, we estimated the standard statistical power, which is the probability that the null hypothesis is rejected at the *α *= 0.05 significance level, without considering the overlap between the detected and real clusters. The random data sets generated under the null model were used to get the critical values of the scan statistics. For *α *= 0.05, this is defined as the 500th highest log likelihood ratio when raning those value from all the 9,999 simulated data sets. The estimated power was then calculated is the proportion of the 1,000 random data sets that had a higher log likelihood ratio than the critical value obtained from the null data sets. The results are shown in Table 3. In general, the cylindrical space-time scan statistic has higher power for the three more compact clusters, while the flexible space-time scan statistic have higher power for the long and narrow the Hudson River cluster. On Day 33 of the high excess risk outbreaks, both methods have very high power.

**Table 3:** Standard power of the prospective space-time scan statistics – flexible and cylindrical – at different days of the outbreak

			Power on Day 31			Power on Day 32			Power on Day 33		
					
Outbreak areas	No. of zip codes s*	excess risk	flex. *K *= 20	cylind. *K *= 20	cylind. 50% pop	flex. *K *= 20	cylind. *K *= 20	cylind. 50% pop	flex. *K *= 20	cylind. *K *= 20	cylind. 50% pop
Cluster A	1	high	0.764	0.860	0.862	0.988	0.996	0.996	0.999	0.999	0.999
Cluster A5	5	high	0.797	0.850	0.847	0.994	0.996	0.996	1.000	1.000	1.000
The Rockaways	5	high	0.769	0.855	0.840	0.992	0.997	0.997	1.000	1.000	1.000
Hudson River	20	high	0.656	0.597	0.632	0.964	0.933	0.949	0.998	0.994	0.995
											
Cluster A	1	med.	0.272	0.357	0.357	0.651	0.733	0.737	0.844	0.915	0.916
Cluster A5	5	med.	0.382	0.435	0.428	0.752	0.801	0.795	0.914	0.940	0.941
The Rockaways	5	med.	0.261	0.373	0.348	0.648	0.768	0.759	0.848	0.924	0.917
Hudson River	20	med.	0.290	0.257	0.297	0.631	0.582	0.610	0.845	0.782	0.803

### Space-time power distribution

In order to compare the performance of the cluster detection tests, the standard power has been derived in the same manner as for usual hypothesis tests. However, it should be noted that standard statistical power reflect the 'power to reject the null hypothesis for whatever reasons,' while the probability of both rejecting the null hypothesis and accurately identifying the true cluster is a different matter altogether.

In order to compare the performance of purely spatial cluster detection tests, Tango and Takahashi [16] proposed a spatial bivariate power distribution *P*_0_(*l*, *s *| s*) based on Monte Carlo simulation where *l *is the length of the significant MLC, while *s *is the number of regions identified out of the true cluster with s* regions.

(6) $$\begin{array}{lll} P_{0}(l,s,|s^{*}) & = & \mathrm{Pr}\{L=l,S=s|s^{*}\}\\ & = & \frac{\#\{\text{significant MLC has length }l\text{ and includes }s\text{ true regions\}}}{\#\{\text{trials for each simulation}\}} \end{array}$$

where *L *and *S *denote the random variable of *l *and *s *under the specified model, respectively, and *l *≥ 1 and 0 ≤ *s *≤ s*. In a similar manner, we propose a space-time *tri*-variate power distribution for a space-time cluster detection test based on Monte Carlo simulation where the temporal length of the true cluster is denoted t*:

(7) $$\begin{array}{l} P_{1}(l,s,t|s^{*},t^{*})=\mathrm{Pr}\{L=l,S=s,U=t|s^{*},t^{*}\}\\ =\frac{\#\{\text{significant MLC has geographical length }l\text{ and includes }s\text{ true regions with temporal length }t\text{\}}}{\#\{\text{trials for each simulation}\}} \end{array}$$

where *U *denotes the random variable of *t *and 1 ≤ *t *≤ *T*.

In Tables 4, 5 and 6, we show the estimated tri-variate power distribution *P*(*l*, *s*, *t *| s*, t*) × 1,000 for (a) Cluster A (s* = 1) on Day 31 (t* = 1) (b) Cluster A5 (s* = 5) on Day 33 (t* = 3) and (c) the Rockaways cluster (s* = 5) on Day 33 (t* = 3), in all cases with high excess risk.

**Table 4:** Space-time power distribution *P*_1_(*l*, *s*, *t *| s*, t*) for the Cluster A (s* = 1) on Day 31 (t* = 1) with high risk (RR= 9. 91), where *t *is a temporal length of detected cluster. The mark "*" is the powers of accurate detection.

(A) flexible (*K *= 20)							
	includes *s *assumed areas						
	0			1			
			
	29-	30-	31-	29-	30-	31-	
length *l *of areas	*t *= 3	2	1	*t *= 3	2	1	total

1	0	0	0	0	6	*315	321
2	0	0	0	0	1	50	51
3	0	0	0	1	2	34	37
4	0	0	0	1	6	34	41
5	0	0	0	2	5	48	55
6	1	0	0	2	2	51	56
7	1	0	0	4	13	35	53
8	0	1	0	1	12	28	42
9	1	1	2	5	6	28	43
10	0	0	0	2	7	22	31
11	1	0	0	1	4	8	14
12	1	1	2	1	0	10	15
13	0	0	0	1	1	2	4
14	0	0	0	0	0	1	1
15	0	0	0	0	0	0	0
16	0	0	0	0	0	0	0
17	0	0	0	0	0	0	0
18	0	0	0	0	0	0	0
19	0	0	0	0	0	0	0
20	0	0	0	0	0	0	0

total	5	3	4	21	65	666	764

(B) cylindrical (*K *= 20)							

	includes *s *assumed areas						
length *l *of areas	0			1			
			
	29-	30-	31-	29-	30-	31-	
	*t *= 3	2	1	*t *= 3	2	1	total

1	0	0	0	5	18	*697	720
2	0	0	0	5	4	63	72
3	0	0	0	1	2	18	21
4	0	0	0	0	4	10	14
5	0	0	0	0	0	2	2
6	1	0	0	1	0	3	5
7	1	0	0	1	1	1	4
8	2	0	0	0	1	1	4
9	0	1	1	0	0	2	4
10	0	0	0	0	0	0	0
11	0	0	0	0	0	1	1
12	0	0	0	0	0	0	0
13	0	0	0	0	0	0	0
14	0	0	0	0	0	2	2
15	0	0	0	0	1	3	4
16	0	0	0	0	0	0	0
17	0	0	0	2	0	1	3
18	1	0	0	0	0	1	2
19	0	0	0	0	0	1	1
20	0	0	0	0	0	1	1

total	5	1	1	15	31	807	860

**Table 5:** Space-time power distribution *P*_1_(*l*, *s*, *t *| s*, t*) for the Cluster A5 (s* = 5) on Day 33 (t* = 3) with high risk (RR = 4. 47), where *t *is a temporal length of detected cluster, and the raw all cells of which have zero powers of both tests is not shown. The mark "*" is the powers of accurate detection.

(A) flexible (*K *= 20)							
	includes *s *assumed areas						
	3		4		5		
				
	31-	32-	31-	32-	31-	32-	
length *l *of areas	*t *= 3	2	*t *= 3	2	*t *= 3	2	total

1							0
2							0
3	12	0					12
4	2	0	74	2			78
5	0	0	37	2	*287	3	329
6	1	0	26	1	158	2	188
7	0	0	16	0	118	2	136
8	0	0	5	0	105	2	112
9	0	0	4	0	67	2	73
10	0	0	6	0	39	1	46
11	0	0	2	0	11	0	13
12	0	0	0	0	10	0	10
13	0	0	1	0	1	0	2
14	0	0	0	0	1	0	1
15	0	0	0	0	0	0	0
16	0	0	0	0	0	0	0
17	0	0	0	0	0	0	0
18	0	0	0	0	0	0	0
19	0	0	0	0	0	0	0
20	0	0	0	0	0	0	0

total	15	0	171	5	797	12	1000

(B) cylindrical (*K *= 20)							

	includes *s *assumed areas						
length *l *of areas	3		4		5		
				
	31-	32-	31-	32-	31-	32-	
	*t *= 3	2	*t *= 3	2	*t *= 3	2	total

1							0
2							0
3	37	1					38
4	2	0	301	7			310
5	2	0	32	0	*0	0	34
6	0	0	5	0	516	10	521
7	0	0	0	0	64	1	65
8	0	0	0	0	5	0	5
9	0	0	0	0	3	0	3
10	0	0	0	0	3	0	3
11	0	0	0	0	4	0	4
12	0	0	0	0	2	0	2
13	0	0	0	0	3	1	4
14	0	0	0	0	1	0	1
15	0	0	0	0	0	0	0
16	0	0	0	0	0	0	0
17	0	0	0	0	0	0	0
18	0	0	0	0	0	0	0
19	0	0	0	0	0	0	0
20	0	0	0	0	0	0	0

total	41	1	338	7	601	12	1000

**Table 6:** Space-time power distribution *P*_1_(*l*, *s*, *t *| s*, t*) for the Rockaways (s* = 5) on Day 33 (t* = 3) with high risk (RR = 8. 48), where *t *is a temporal length of detected cluster, and the raw all cells of which have zero powers of both tests is not shown. The mark "*" is the powers of accurate detection.

(A) flexible (*K *= 20)											
	includes *s *assumed areas										
	1	2	3		4			5			
						
	31-	31-	31-	32-	31-	32-	33	31-	32-	33	
length *l *of areas	*t *= 3	*t *= 3	*t *= 3	2	*t *= 3	2	1	*t *= 3	2	1	total

1	0										0
2	0	0									6
3	0	2	6	0							22
4	0	0	20	0	181	1	0				204
5	0	0	22	0	50	0	0	*571	2	0	626
6	0	0	3	0	26	1	0	23	1	0	52
7	0	0	1	0	9	0	0	54	1	1	66
8	0	0	1	0	2	0	0	11	0	0	14
9	0	0	1	0	2	0	0	6	0	0	8
10	0	0	0	0	0	0	0	1	0	0	1
11	0	0	0	0	0	0	0	1	0	0	1
12	0	0	0	0	0	0	0	0	0	0	0
13	0	0	0	0	0	0	0	0	0	0	0
14	0	0	0	0	0	0	0	0	0	0	0
15	0	0	0	0	0	0	0	0	0	0	0
16	0	0	0	0	0	0	0	0	0	0	0
17	0	0	0	0	0	0	0	0	0	0	0
18	0	0	0	0	0	0	0	0	0	0	0
19	0	0	0	0	0	0	0	0	0	0	0
20	0	0	0	0	0	0	0	0	0	0	0

total	0	8	48	0	270	2	0	667	4	1	1000

(B) cylindrical (*K *= 20)											

	includes *s *assumed areas										
length *l *of areas	1	2	3		4			5			
						
	31-	31-	31-	32-	31-	32-	33	31-	32-	33	
	*t *= 3	*t *= 3	*t *= 3	2	*t *= 3	2	1	*t *= 3	2	1	total

1	2										2
2	0	8									8
3	0	0	52	1							53
4	0	0	0	0	876	6	1				883
5	0	0	1	0	3	0	0	*0	0	0	4
6	0	0	0	0	32	0	0	0	0	0	32
7	0	0	0	0	14	1	0	1	0	0	16
8	0	0	0	0	2	0	0	0	0	0	2
9	0	0	0	0	0	0	0	0	0	0	0
10	0	0	0	0	0	0	0	0	0	0	0
11	0	0	0	0	0	0	0	0	0	0	0
12	0	0	0	0	0	0	0	0	0	0	0
13	0	0	0	0	0	0	0	0	0	0	0
14	0	0	0	0	0	0	0	0	0	0	0
15	0	0	0	0	0	0	0	0	0	0	0
16	0	0	0	0	0	0	0	0	0	0	0
17	0	0	0	0	0	0	0	0	0	0	0
18	0	0	0	0	0	0	0	0	0	0	0
19	0	0	0	0	0	0	0	0	0	0	0
20	0	0	0	0	0	0	0	0	0	0	0

total	2	8	53	1	927	7	1	1	0	0	1000

This tri-variate power distribution provides us with a detailed description of the space-time cluster detection tests performance. For the outbreak in cluster A with a single ZIP code, the cylindrical scan statistic has higher power to detect the cluster with complete accuracy, with *P*_1_(*l *= 1, *s *= 1, *t *= 1 | s*, t*) = 697/1000, compared to 315/1000 for the flexible. Moreover, the flexible scan statistic has a heavier tail in the (*s*, *t*) = (1, 3) column than the cylindrical one. However the cylindrical scan detected some large clusters including several with *l *≥ 15. For outbreaks in the non-circular shaped A5 and Rockaway clusters, the flexible scan statistic has higher power for complete accurate detection. Indeed, the cylindrical scan statistic cannot detect these clusters with complete accuracy since they are not circular, so that the power for complete accuracy is zero. Moreover, note that for cluster A5, the flexible scan statistic is more likely to include all the five areas in the true cluster (797 + 12 = 809/1000 versus 601 + 12 = 613/1000), and it is also more likely to avoid including any of the ZIP codes outside the true cluster (12 + 74 + 2 + 287 + 3 = 378/1000 versus 37 + 1 + 301 + 7 = 346/1000). For the Rockaway cluster, the flexible scan statistic is again more likely to include all the five areas in the true cluster (667 + 4 + 1 = 672 versus 1 + 0 + 1 = 1), but the cylindrical scan statistic avoids the ZIP codes outside the cluster more often (2 + 8 + 52 + 1 + 876 + 6 + 1 + 0 + 0 + 0 = 946/1000 versus 0 + 0 + 6 + 0 + 181 + 1 + 0 + 571 + 2 + 0 = 761/1000). Tables 5 and 6 show that the temporal accuracy of the detected cluster is very good for both methods. For example, for cluster A5, the flexible scan has *P*_1_(+, +, 3 | s*, t*) = ∑_*l *_∑_*s*_*P*_1_(*l*, *s*, 3 | s*, t*) = (15 + 171 + 797)/1000 = 0.983 while the cylindrical scan has *P*_1_(+, +, 3 | s*, t*) = (41 + 338 + 601)/1000 = 0.980.

The complexity of the three-dimensional tri-variate power distributions suggests that we need some summary measure. Since the temporal accuracy is very similar, we focus on the geographical accuracy. We will compute the extended power of spatial cluster detection tests, as developed by Takahashi and Tango [26]. We will also define and compute geographical sensitivity and false positive rates.

#### The extended power

We can consider two types of spatial misclassifications when applying the cluster detection test (CDT). One is a *false negative test result *(FN) in which the CDT misses a region included in the true cluster. Sensitivity is 1 - FN rate. The other is a *false positive test result *(FP) in which the CDT incorrectly detects a region that is not present in the true cluster. The numbers of FNs and FPs for geographical detection are s* - *s *and *l *- *s*, respectively.

The extended power is based on the bivariate distribution *P*_0_(*l*, *s *| s*) and penalties introduced for the FPs and FNs of the geographical detection as

(8) $$I(w^{-},w^{+})=\sum_{l\geq 1}\sum_{s\geq 0}W(l,s;w^{-},w^{+})P_{0}(l,s|s^{*})$$

where *W*(*l*, *s*; *w*^-^, *w*^+^) is a weight function such that

(9) $$W(l,s;w^{-},w^{+})=\{\begin{array}{ll} \sqrt{\left(1-\min\{w^{-}(s^{*}-s),1\}\right)\left(1-\min\{w^{+}(l-s),1\}\right),} & \\ & (s\leq l;0\leq s\leq s^{*},1\leq l),\\ 0, & \left(\text{otherwise}\right) \end{array}$$

and *w*^- ^and *w*^+ ^are the predefined penalties for the FNs and FPs (per region), respectively. This power includes the following three special powers:

1. The standard power as *I*(0, 0).

2. The power to detect the geographical true cluster accurately as *I*(1, 1).

3. The power for which the MLC includes all the regions within the true cluster as *I*(1, 0).

Takahashi and Tango [26] also proposed the profile of the extended power as

**Figure 3:**
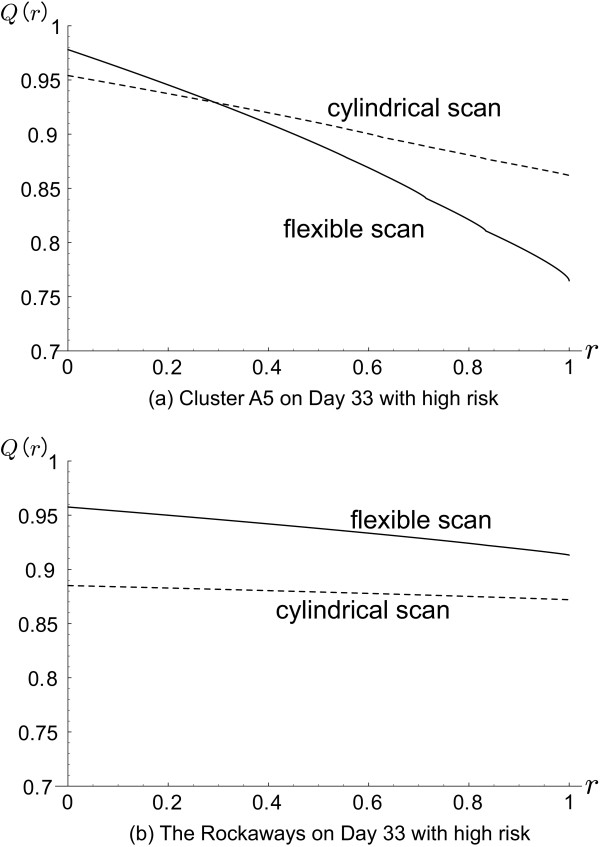
Profile of the extended power *Q*(*r *| s*) for flexible and cylindrical scan statistics applied to the cluster (a) Cluster A5, and (b) The Rockaways.

(10) *Q*(*r *| s*) = *I*(1/s*, r/s*),     (0 ≤ *r *≤ 1)

where *r *= *w*^+^/*w*^- ^with *w*^- ^= 1/s*, because it is difficult to set the value of *w*^- ^and *w*^+ ^in advance. Figure 3 shows the plots of the profile *Q*(*r *| s*) against *r *(0 ≤ *r *≤ 1) for flexible and cylindrical scan statistics applied to (a) the cluster A5 and (b) the Rockaways, both on Day 33 with high risk, based upon Tables 5 and 6. Figure 3(a) shows the flexible scan statistic has higher extended power when *r *= 0 i.e. penalties for the FP *w*^+ ^= 0, *I*(1/5, 0) = 0.978 for the flexible and 0.954 for the cylindrical, while the extended power of cylindrical scan statistic is higher for large *r*, as *I*(1/5, 1/5) = 0.765 for the flexible and 0.862 for the cylindrical. On the other hand, Figure 3(b) shows the flexible scan statistic is more uniformly powerful than the cylindrical one for the Rockaways cluster, *I*(1/5, 0) = 0.958 and *I*(1/5, 1/5) = 0.913 for the flexible, and *I*(1/5, 0) = 0.885 and *I*(1/5, 1/5) = 0.872 for the cylindrical, respectively.

#### Sensitivity and positive predictive value

As other measures of accuracy of cluster detection tests, we shall consider sensitivity and positive predictive value [27,28]. These measures can be defined in terms of either *the number of regions *or *the population*. First, we define *sensitivity *of cluster detection tests as the probability of detecting the regions that actually constitute the cluster, i.e, proportion of the number of regions correctly detected from the true cluster, s/s*. We shall present the expected value:

(11) $$TP_{1}=E\left[\frac{S}{s^{*}}\right]=\sum_{s=0}^{s^{*}}\frac{s}{s^{*}}P_{0}(+,s|s^{*}).$$

*Positive predictive value *(PPV) of cluster detection tests is defined in a similar manner as the proportion of the number of true regions in the detected cluster, i.e, *s/l *under *l *> 0, and the expected value is presented:

(12) $$PP_{1}=E\left[\frac{S}{L}|L>0\right]=\sum_{l\geq 1}\sum_{s\geq 0}\frac{s}{l}\frac{P_{0}(l,s|s^{*})}{P_{0}(+,+|s^{*})}$$

Based upon the population, we can define the following sensitivity *TP*_2 _and positive predictive value *PP*_2_:

(13) $$TP_{2}=\frac{E(\text{Total population of the detected }S\text{ true regions})}{\text{Total population of the }s^{*}\text{ reigons of true cluster}}$$

(14) $$PP_{2}=E\left(\frac{\text{Total population of }S\text{ true regions}}{\text{Total population of }L\text{ detected regions}}|L>0\right)$$

All these summary measures are better the larger they are with 100 being the optimal.

Table 7 shows the sensitivity and PPV of the flexible and cylindrical space-time scan statistics for each cluster with a high relative risk. For cluster A, the cylindrical scan statistic has higher PPV and higher sensitivity than the flexible one. For cluster A5 and the cylindrical has higher PPV on all days and higher sensitivity on day 31, but the flexible scan statistic has higher sensitivity on days 32 and 33. The same is true for the Rockaway cluster. For the Hudson River cluster, the flexible scan statistic has higher PPV than the cylindrical. The flexible scan has higher sensitivity than for the cylindrical with the same upper constant *K *= 20 on the number of regions in the detected cluster, but lower sensitivity compared to the cylindrical scan with a 50% upper limit on the cluster size. Note though, that this difference in sensitivity is less than the difference in PPV that goes the other way.

**Table 7:** Sensitivity and positive predictive value (PPV) of the flexible and cylindrical space-time scan statistics.

			zip codes		population	
		traditional power	sensitivity (%)	PPV (%)	sensitivity (%)	PPV (%)
**Cluster A**; s* = 1; high risk						
Day 31	flexible (*K *= 20)					
	cylindrical (*K *= 20)	0.860	85.30	89.45	85.30	91.50
	cylindrical (50% pop)	0.862	85.90	88.79	85.90	90.84
Day 32	flexible (*K *= 20)	0.988	98.80	84.50	98.80	87.80
	cylindrical (*K *= 20)	0.996	99.50	97.44	99.50	98.18
	cylindrical (50% pop)	0.996	99.50	97.33	99.50	98.08
Day 33	flexible (*K *= 20)	0.999	99.90	96.27	99.90	97.32
	cylindrical (*K *= 20)	0.999	99.90	99.48	99.90	99.65
	cylindrical (50% pop)	0.999	99.90	99.48	99.90	99.65
						
**Cluster A5**; s* = 5; high risk						
Day 31	flexible (*K *= 20)	0.797	66.08	55.93	67.29	63.00
	cylindrical (*K *= 20)	0.850	69.62	80.35	71.65	84.21
	cylindrical (50% pop)	0.847	70.62	78.17	71.62	82.02
Day 32	flexible (*K *= 20)	0.994	92.22	70.17	92.94	76.73
	cylindrical (*K *= 20)	0.996	88.78	85.14	90.86	89.41
	cylindrical (50% pop)	0.996	88.96	84.81	91.05	89.11
Day 33	flexible (*K *= 20)	1.000	95.88	80.02	96.64	85.25
	cylindrical (*K *= 20)	1.000	91.42	87.32	93.66	91.67
	cylindrical (50% pop)	1.000	91.42	87.30	93.66	91.65
						
**The Rockaways**; s* = 5; high risk						
Day 31	flexible (*K *= 20)	0.769	60.68	72.09	69.04	73.58
	cylindrical (*K *= 20)	0.855	62.32	91.63	74.45	91.76
	cylindrical (50% pop)	0.840	61.40	91.04	73.65	91.15
Day 32	flexible (*K *= 20)	0.992	86.76	87.36	94.17	89.86
	cylindrical (*K *= 20)	0.997	77.00	96.84	92.75	97.46
	cylindrical (50% pop)	0.997	77.00	96.84	92.75	97.46
Day 33	flexible (*K *= 20)	1.000	92.16	93.81	97.15	95.97
	cylindrical (*K *= 20)	1.000	78.50	98.06	94.51	98.59
	cylindrical (50% pop)	1.000	78.50	98.06	94.51	98.59
						
**Hudson River**, s* = 20; high risk						
Day 31	flexible (*K *= 20)	0.656	20.07	64.99	26.00	69.72
	cylindrical (*K *= 20)	0.597	14.23	61.10	18.16	65.18
	cylindrical (50% pop)	0.632	26.15	50.70	31.26	53.71
Day 32	flexible (*K *= 20)	0.964	32.17	73.59	41.81	78.36
	cylindrical (*K *= 20)	0.933	24.13	61.55	31.58	66.69
	cylindrical (50% pop)	0.949	42.90	50.27	51.50	53.96
Day 33	flexible (*K *= 20)	0.998	34.91	79.39	46.27	84.17
	cylindrical (*K *= 20)	0.994	27.23	60.56	36.75	66.20
	cylindrical (50% pop)	0.995	48.14	47.34	58.54	51.34

## Conclusion

In this paper, we have proposed a flexible space-time scan statistic to detect arbitrarily shaped disease outbreaks. We have also presented a tri-variate power distribution which is useful for evaluating the performance of cluster detection tests, informing us about the spatial and temporal accuracy of the detected clusters in addition to the standard statistical power.

For the benchmark data evaluated in this paper, the cylindrical scan statistic performs better for the small single zip-code cluster, although by the third day of the outbreak both methods are almost perfect. For the small irregular shaped clusters, A5 and Rockaways, the cylindrical performs better on the first day of the outbreak, but as more data accumulates, the flexible scan statistic has certain advantages in determining the precise size and shape of the outbreak. For the large and narrow Hudson River cluster, the flexible scan statistic performs better than the cylindrical one, with slightly higher standard power, much higher PPV and slightly higher or lower sensitivity depending on the type of cylindrical method used. Results may be different for other types of regular and irregularly shaped disease outbreaks, but the four examples used in this paper gives some sense of the proposed methods performance.

For early detection, timeliness is much more important than geographical accuracy. When monitoring an occurring outbreak, on the other hand, geographical accuracy becomes critical and is then the key objective since we already know the outbreak is there. Our results suggest that we may use both the cylindrical and flexible scan statistic for disease outbreak detection, but for different purposes. Specifically, for detecting new outbreak that, one may want to use the cylindrical scan statistic. That is especially if we expect the outbreak to start locally, within a reasonably small and compact area containing only a few ZIP-codes. On the other hand, once the outbreak has spread to a larger area, and we want to monitor that spread, one may want to use the flexible scan statistic, with its ability to accuratly determine the precise geographical extent of irregular shaped outbreaks. This is especially true ones the outbreak has left its local area of origin.

To evaluate the performance of space-time scan statistic, we applied the extended power for purely spatial cluster detection test (8), which is defined as the weighted sum of the bivariate power distribution wherein the weight is given by the geometric mean of (1-penalty for the false negatives) and (1-penalty for the false positives), including the standard power as a special case. Also we applied the profile *Q*(r | s*) proposed by Takahashi and Tango [26]. This plot gave us a detailed description regarding power of cluster detection tests. Needless to say, it is possible to extend it to space-time version if we could consider the penalties for temporal false negatives and false positives, but we leave this problem for future work. Also, for the profile of the extended power, we chose to use a fixed cost of *w*^- ^= 1/s* for false negatives and a smaller or equal cost for false positives. For more general situations, we could plot the full bivariate extended power function on the unit square.

Similarly to the flexible spatial scan statistic in the purely spatial situation, the flexible space-time scan statistics proposed in this paper has a limitation of cluster size, because of the limitation of the speed of computation. The proposed scan statistic works well for small to moderate sized clusters. Although we set the maximum length of the geographical window to *K *= 20, this is not large enough to detect the 20 ZIP codes of the Hudson River cluster accurately because this cluster is too long to be the subset of the 20-th nearest neighbors of any region. Computation time depends on the size of the data set and *K*. Indeed, for the August 11 analysis of respiratory syndrome data in Massachusetts, with 385 ZIP codes, a maximum temporal length of *T *= 7 days, a maximum spatial size of *K *= 20, and with 999 Monte Carlo replications, the flexible space-time scan statistic took 87.7 minutes to run on a 3.06-GHz Pentium 4 computer, while the cylindrical space-time scan statistic took only 9.8 minutes.

A limitation of length may also prevent the analysis to present large clusters of unlikely and very peculiar shapes. These undesirable properties produced by maximum likelihood ratio might suggest the use of different criterion for model selection, including some penalized likelihood [20,29]. Also, for larger cluster seizes, the method is not practically feasible and a more efficient algorithm is needed.

In this paper, we considered the *right *cylinder or *right *prism of the cluster model, as an expansion of the cylindrical space-time scan statistic for a prospective disease surveillance by Kulldorff [10]. This does not allow the scanning window to adjust itself as the disease outbreak grows or shrinks geographically over time. Recently, Iyengar has suggested using a *square pyramid shape *window which can model either growth (or shrinkage) and movement of the disease cluster [30]. For the proposed flexible space-time scan statistic, if we could consider the flexibility in both space and time, that is, evaluating all connected subsets within a cylinder instead of W in (4), we can detect more arbitrarily shaped clusters in space-time. For such an expansion, an efficient computational algorithm will be needed for the scanning process, as well as a more sophisticated mechanism for the interpretation of such complicatedly shaped clusters. The implementation and importance of such methods for disease surveillance and monitoring, is an issue for future research.

## Authors' contributions

KT, MK and TT developed the statistical methodology and designed the study. KT, MK and KY analyzed and interpreted the syndromic surveillance data. KT programmed the methods, did the power calculations and wrote the first draft of the manuscript. All authors participated in the interpretation of the results, revised the manuscript, and approved the final version.
